# The small genome segment of Bunyamwera orthobunyavirus harbours a single transcription-termination signal

**DOI:** 10.1099/vir.0.042390-0

**Published:** 2012-07

**Authors:** Gjon Blakqori, Anice C. Lowen, Richard M. Elliott

**Affiliations:** Biomedical Sciences Research Complex, School of Biology, University of St. Andrews, North Haugh, St. Andrews KY16 9ST, Scotland, UK

## Abstract

Transcription termination of the mRNA produced from the small (S) genome segment of Bunyamwera orthobunyavirus (BUNV) has previously been mapped to two *cis*-acting sequences located within the 5′ UTR using a virus-free replication assay. The ability of these sequences to terminate transcription was attributed to the shared pentanucleotide motif 3′-UGUCG-5′. Taking advantage of our plasmid-based rescue system to generate recombinant viruses, we re-evaluated the importance of both pentanucleotide motifs as well as that of two other conserved sequences in transcription termination *in vivo*. Analysis of the 3′ ends of positive-stranded viral RNAs derived from the S segment revealed that only the region around the upstream pentanucleotide motif mediated transcription termination in cells infected with wild-type BUNV, leading to mRNAs that were about 100 nt shorter than antigenome RNA. Furthermore, the downstream motif was not recognized in recombinant viruses in which the upstream signal has been disrupted. Our results suggest that in the context of virus infection transcription termination of the BUNV S genome segment mRNA is exclusively directed by the upstream-termination signal. Interestingly, within this region we identified a motif similar to a transcription-termination sequence used by Rift Valley fever phlebovirus.

## Introduction

Bunyamwera virus (BUNV; family *Bunyaviridae*, genus *Orthobunyavirus*) is a negative-strand RNA virus with a tri-segmented genome comprising small (S), medium (M) and large (L) segments. Each of the segments consists of a coding region that is flanked by 3′ and 5′ UTRs. The UTRs are multi-functional as they contain the promoters that regulate transcription and replication of the viral genome, provide a signal for encapsidation by the nucleocapsid (N) protein and are implicated in the packaging of encapsidated genomes into virions (Dunn *et al.*, 1995; Flick & Pettersson, 2001; Osborne & Elliott, 2000; Weber *et al.*, 2001). The L segment encodes the viral polymerase (L) and the M segment expresses a polyprotein that is co-translationally cleaved into the virion glycoproteins Gn and Gc as well as the non-structural protein NSm (Elliott, 1989b; Fazakerley *et al.*, 1988). The BUNV S segment encodes two proteins, N, and the non-structural protein NSs, which are translated from overlapping reading frames in the same mRNA (Dunn *et al.*, 1994; Elliott, 1989a). Synthesis of orthobunyavirus mRNAs starts from a short, 5′-capped primer, which is acquired from host cell mRNAs by means of cap-snatching (Bouloy & Hannoun, 1976; Eshita *et al.*, 1985; Patterson *et al.*, 1984). Transcription terminates before the polymerase reaches the end of the genomic template, leading to mRNAs that have truncated 3′ ends and furthermore appear to be non-polyadenylated (Abraham & Pattnaik, 1983; Bouloy *et al.*, 1984; Elliott, 1985; Pattnaik & Abraham, 1983). The 3′ UTR of the BUNV S segment-derived mRNA contains a sequence, dubbed the translation-enhancing element (TEE), that promotes efficient translation of viral mRNA in a poly(A) binding protein independent manner (Blakqori *et al.*, 2009). While L and M segment mRNAs are shortened by about 40 nt, the S segment mRNA lacks approximately 100 nt (Bouloy *et al.*, 1990; Cunningham & Szilágyi, 1987; Eshita *et al.*, 1985; Jin & Elliott, 1993; Patterson *et al.*, 1984). The exact mechanism of how and why the viral L polymerase concludes transcription at these sites is currently unknown, but is expected to be different from that of many other segmented and non-segmented negative-strand RNA viruses which terminate at A/U-rich sequences and encode short poly(U) stretches that mediate polyadenylation through reiterative transcription (Li & Palese, 1994; Whelan *et al.*, 2004).

**Fig. 1.**
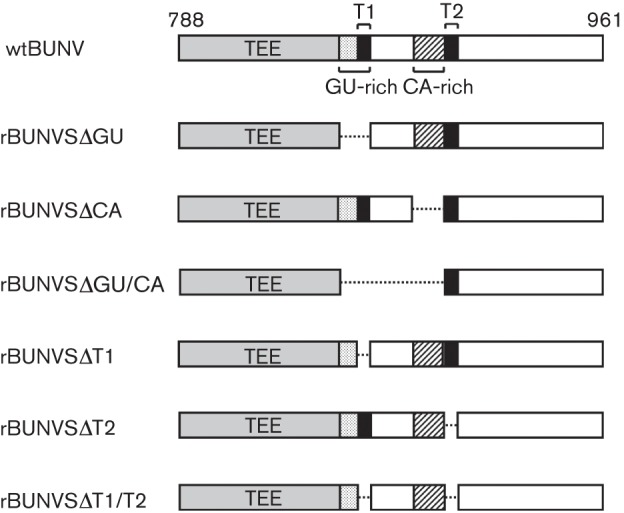
Overview of the deletions in the genomic 5′ UTR (nt 788–961) of the S segments of recombinant viruses rescued in this work. The designations correspond to the following nucleotide deletions: ΔGU: 850–868, ΔCA: 873–895, ΔGU/CA: 850–895, ΔT1 : 865–870, ΔT2 : 898–902, ΔT1/ΔT2 : 865–870 and 898–902. TEE encompassing nt 788–861 (Blakqori *et al.*, 2009).

Alignment of the 5′ UTRs of orthobunyavirus S segments revealed two conserved sequence motifs proximal to the site of transcription termination, a GU-rich region and a CA-rich region (Dunn *et al.*, 1994). Furthermore, another study identified two sequences within the S segment 5′ UTR, designated T1 and T2, that were implicated in transcription termination in a virus-free minireplicon system (Barr *et al.*, 2006). A six nucleotide sequence in T1 (3′-GUCGAG-5′) was demonstrated to play a significant role in termination, and this sequence overlapped a pentanucleotide motif (3′-UGUCG-5′) shared between T1 and T2. This pentanucleotide is also present in the 5′ UTR of the BUNV L segment. The authors suggested that the pentanucleotide motif is crucial for transcription termination, with surrounding nucleotides contributing to the termination signal. Although both sequences had the potential to direct termination, the downstream signal (T2) was only recognized in the absence of the upstream signal (T1), implying some degree of hierarchy. While the T1 pentanucleotide motif is located within the GU-rich conserved region, the T2 motif lies downstream of the conserved CA-rich region (see Fig. 1). To investigate the roles of these sequences in transcription termination in the context of virus infection we generated a series of recombinant BUNV containing specific deletions in their S genome segments. Our data indicate that mRNA transcription terminates almost exclusively at the upstream signal.

## Results and Discussion

### Rescue of recombinant viruses and initial characterization in cell culture

We constructed S segment cDNA clones that omitted the various motifs (Fig. 1) and used these to generate recombinant viruses by our previously described three-plasmid rescue system (Lowen *et al.*, 2004). Rescue was successful for all constructs, demonstrating that none of the deleted sequences are essential for virus viability. Previously, S segment cDNA clones with larger deletions in the genomic 5′ UTR that encompassed most or all of the described motifs failed to produce viable virus (Lowen & Elliott, 2005). The recombinant viruses created in this work were designated rBUNVSΔGU, rBUNVSΔCA, rBUNVSΔGU/CA, rBUNVSΔT1, rBUNVSΔT2 and rBUNVSΔT1/T2, indicating the deleted motifs (see Fig. 1 for details). All genomic alterations were confirmed by nucleotide sequencing of S segments that were recovered from infected cells by means of RT-PCR.

**Fig. 2.**
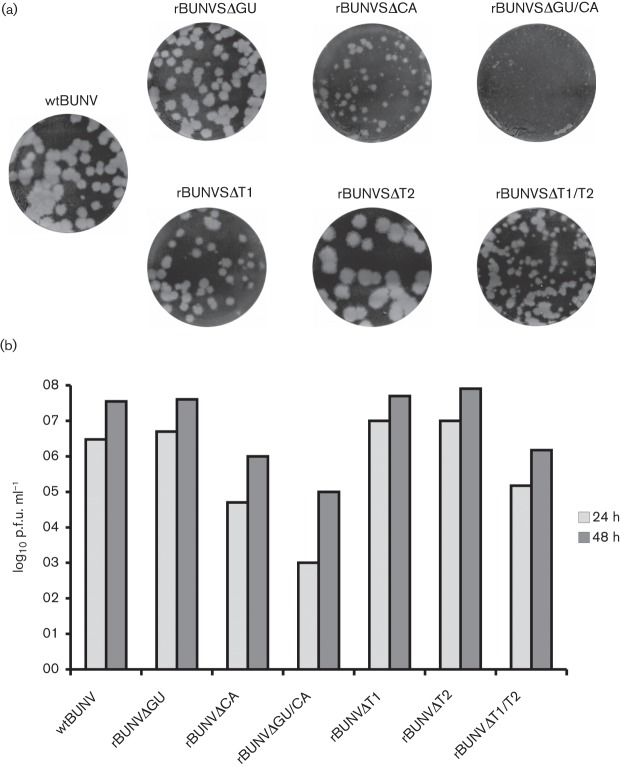
Growth phenotype in cell culture. (a) Plaque phenotype of recombinant viruses in Vero cells. (b) Multi-step growth on BHK-21 cells. Cells were infected at an m.o.i. of 0.01 and titres in supernatants were determined by plaque assay at the indicated time points post-infection. Representative results of two experiments are shown.

For initial characterization, multi-step growth kinetics of the newly generated viruses were monitored in baby hamster kidney (BHK-21) cells. Cells were infected at an m.o.i. of 0.01 and supernatants collected 24 and 48 h post-infection. The amount of infectious virus in the supernatant was determined by plaque assay. The recombinant viruses exhibited different phenotypes in cell culture and could roughly be categorized into three classes (Fig. 2). Most viruses with deletion of a single region, namely rBUNVSΔGU, rBUNVSΔT1 and rBUNVSΔT2, displayed wild-type (wt)-like growth, and their plaque sizes were also comparable to wtBUNV. Moderate attenuation was observed for two viruses, rBUNVSΔCA and rBUNVSΔT1/T2, which displayed a 100-fold decrease in titre after 48 h and a smaller plaque phenotype. Lastly, rBUNVSΔGU/CA grew to 1000-fold lower titres as compared with wtBUNV and produced pinhead-sized plaques. Thus, the main determinant for fitness in cell culture, from the sequences investigated in this work, appeared to be the conserved CA-rich region, which does not contain either of the two previously described pentanucleotide motifs.

**Fig. 3.**
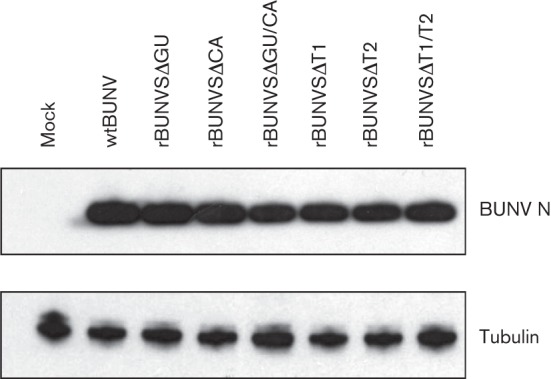
N protein production. BHK-21 cells were infected at an m.o.i. of 0.01 and cells were lysed 48 h post-infection. Lysates were separated by electrophoresis and blotted proteins were detected using antibodies against BUNV N and tubulin proteins.

We next examined viral protein production to determine whether the omission of sequences in the 5′ UTR had a detrimental effect on S segment gene expression. BHK-21 cells were infected at an m.o.i. of 0.01, lysed 48 h later and lysates were subjected to Western blotting using a monospecific antibody against the viral N protein. As shown in Fig. 3, all viruses accumulated similar amounts of N protein over the observed period of time, indicating that the reduced plaque size and lower titres that were evident in some of the recombinant viruses is likely not due to a bottleneck in protein production.

### Mapping of the S segment transcription-termination sites

**Fig. 4.**
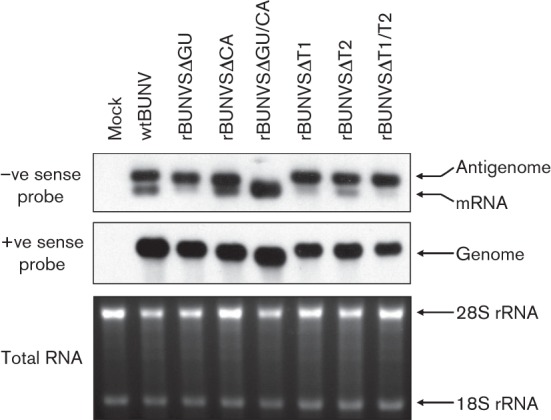
RNA profiles of virus-infected cells. Northern blot assay showing RNA species detected with −ve sense (upper panel) and +ve sense (middle panel) S segment probes, respectively. Positions of antigenome, mRNA and genome RNA are indicated. Lower panel: loading control of ethidium bromide-stained total RNA before blotting of the +ve sense-blot. Positions of 28S and 18S rRNAs are indicated.

Northern blot analysis of infected cell RNA was conducted to assess the role of the deleted sequences in transcription termination. Genomic and antigenomic RNAs were detected using digoxigenin-labelled probes complementary to either the genome (+ve sense probe) or the antigenome and mRNA (−ve sense probe) of the BUNV S segment. As shown in Fig. 4, the −ve sense probe detected two RNA species in cells infected with wtBUNV, the slower migrating band representing the antigenome (961 nt) and the faster migrating band corresponding to the S segment mRNA. This pattern was conserved in viruses in which either the T2 pentanucleotide motif or the CA-rich region was deleted, but contained an intact T1-termination site, demonstrating that they were dispensable for termination at T1. Strikingly, the mRNA band was absent from cells infected with viruses that lacked the intact T1 pentanucleotide motif, namely rBUNVSΔT1, rBUNVSΔT1/T2 and rBUNVSΔGU, indicating transcription read-through to the end of the template. For virus rBUNVSΔGU/CA, we observed a single, faster migrating broad band on the blot (the S segment of this virus contains a 45 nt deletion). Since this virus also lacked the T1 pentanucleotide motif we assumed that there is read-through leading to RNA of uniform length. Northern blot analysis using the +ve sense probe revealed that almost all recombinant viruses produced S genomes to levels comparable to that of wt virus (Fig. 4). Although there was slightly less genomic RNA in cells infected with recombinant virus rBUNVSΔT1/T2, the same was observed for viruses rBUNVSΔT1 and rBUNVSΔT2 that displayed almost wt-like fitness in cell culture. Thus, the amounts of RNA did not seem low enough to explain a 99 % drop in production of infectious virus particles. Taken together, the Northern blot results suggested that partial or complete deletion of the T1 pentanucleotide motif always led to the occurrence of a single band corresponding to the size of antigenomic S RNA, indicating read-through of the transcription-termination signal.

**Fig. 5.**
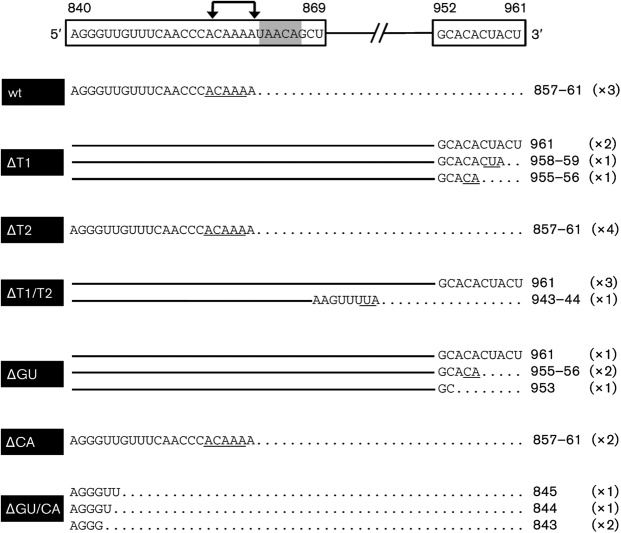
Mapping of the 3′ end of positive-stranded viral RNAs. The 3′ ends of wt and recombinant BUNV RNAs were determined by nucleotide sequencing of TA-cloned 3′-RACE products. Depicted at the top is a schematic representation of the 3′ UTR of the BUNV S antigenomic RNA (nt 840–961). Nucleotide sequences of the regions with termination hot spots (840–868 and 952–961) are in boxes. For each clone the sequence is aligned to the schematic on top, followed by either the number of the 3′ nucleotide or the region at which termination could have occurred (and which is underlined in the sequence). The number in parentheses indicates how often an identical sequence was identified.

In order to determine the exact termini of the S segment transcripts, total RNA of infected cells was isolated and subjected to rapid amplification of 3′ ends (3′ RACE). Amplified products were cloned and multiple clones of each virus-infected cell sample sequenced (Fig. 5). This analysis revealed that for viruses with an intact T1-termination signal (wtBUNV, rBUNVSΔT2 and rBUNVSΔCA) transcription predominantly concludes at nt 857–861 (9 of 9 clones), two to six nucleotides upstream of the T1 pentanucleotide motif. Note that because of the A-tailing step in the 3′ RACE protocol, mRNAs could terminate opposite the G residue at position 857 or any of the next four U residues. In agreement with the Northern blotting results, lack of the T1 motif (viruses rBUNVSΔT1, rBUNVSΔT1/T2 and rBUNVSΔGU) led to read-through of the transcription-termination signal, resulting in full-length, or close to full-length, RNAs.

A notable exception to the observed pattern of termination at T1 or complete read-through was virus rBUNVSΔGU/CA, which produced shorter transcripts 843–845 nt in length. This virus seems to synthesize an abundance of subgenomic RNAs, explaining the broad band that was observed for both positive- as well as negative-stranded RNA in Northern blotting (Fig. 4). As neither of the viruses with a single deletion of these regions, rBUNVSΔGU or rBUNVSΔCA, exhibited the propensity for premature termination, the GU-rich and CA-rich sequences might co-operatively act in regulating transcription and/or replication. The fact that none of the 3′ RACE clones contained a full-length antigenomic sequence suggests that read-through is a rare event in this virus and it is conceivable that the attenuated growth is due to a shortage of full-length antigenomic RNA, and hence genomic S segments.

### The T1 pentanucleotide motif overlaps with an alternative termination site

An earlier study mapped the 3′ end of the S mRNA to be in the region of nt 851–861 (Jin & Elliott, 1993). Here, we show that the majority of S segment mRNAs indeed terminate between nt 857 and 861. Furthermore, we could confirm that nt 865–870 are essential for termination at this site (Barr *et al.*, 2006). By contrast, the second pentanucleotide motif, as well as the CA-rich region, were both redundant for termination at T1. We also found no evidence in virus-infected cells that the T2 pentanucleotide motif was capable of transcription termination downstream of T1, independent of the presence or absence of T1. Thus, our *in vivo* data are at variance with the previous finding that termination can occur at a second site around the T2 pentanucleotide motif if the T1 signal was disrupted (Barr *et al.*, 2006). Several methodical differences between our study and that of Barr *et al.* could account for the discrepancies concerning the second termination signal. Firstly, Barr *et al.* utilized a minireplicon system in which primary transcription from transfected cDNA plasmids is accomplished by T7 DNA-dependent RNA polymerase, followed by transcription and replication of the minireplicon RNA by the viral L polymerase (Dunn *et al.*, 1995). By contrast, we produced recombinant viruses in which transcription and replication are exclusively facilitated by the L polymerase. Secondly, while we introduced nucleotide deletions, Barr *et al.* mainly scrambled nucleotides stretches in order to disrupt sequences. It is conceivable that nucleotide scrambling put the second pentanucleotide motif in a context that favours recognition by the viral polymerase and that transcription termination directed by this motif was related to artificial experimental conditions. Alternatively, termination at T2 might have occurred at a rate that was too low for the resulting transcripts to be identified in our assays, and more sensitive approaches such as deep sequencing might detect rare RNA species.

**Fig. 6.**
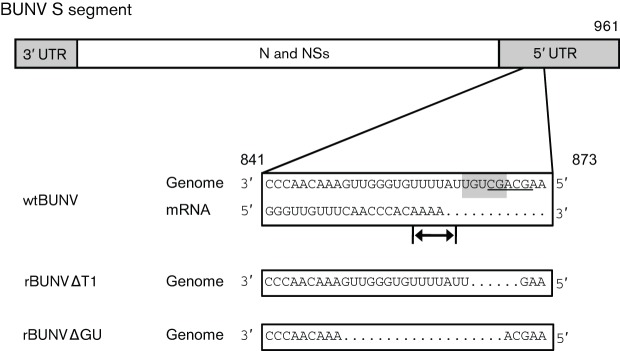
The BUNV S segment harbours an RVFV-like termination signal. Schematic of the BUNV S segment, highlighting the originally described T1 transcription-termination sequence (nt 841–873) in genomic (−ve) sense, and the sequence of the mRNA 3′ end (+ve sense). The previously described pentanucleotide (Barr *et al.*, 2006) is shaded, and the RVFV-like pentanucleotide is underlined. The 3′ end of the mRNA is in the region of nt 857–861 as indicated by arrows. Deletions in the S genomes of recombinant viruses rBUNVΔT1 and rBUNVΔGU are shown below.

In any case, the obvious differences in the ability of sites T1 and T2 to direct termination indicates that termination probably depends additionally on nucleotides that surround the pentanucleotide motifs. Indeed, the BUNV S-termination signal was originally mapped to a 33 nt stretch covering nt 841–873, with residues other than the core 865–870 motif ‘possessing individually minor, but collectively significant, signalling ability’ (Barr *et al.*, 2006; Fig. 6). Studies on Rift Valley fever virus (RVFV; in the genus *Phlebovirus* of the family *Bunyaviridae*) showed that mRNA transcription terminates at the conserved sequence 3′-CG**G-5′, wherein position 3 is either A or U and position 4 either C or U (Albariño *et al.*, 2007; Ikegami *et al.*, 2007; Lara *et al.*, 2011). Similar termination sequences have been determined experimentally for Toscana and sandfly fever Sicilian phleboviruses, and are also predicted in the genomes of Uukuniemi and Punta Toro phleboviruses (Albariño *et al.*, 2007), though data for other bunyaviruses are lacking (Walter & Barr, 2011). The sequence 3′-CGACG-5′ is present in the BUNV S segment, nt 867–871, and partly overlaps the previously identified pentanucleotide that is shared between T1 and T2 (Fig. 6). The sequence 3′-CGACG-5′ is recognized by the RVFV L polymerase and directs termination at a site three to five nucleotides upstream of its location in the template RNA (Lara *et al.*, 2011). Intriguingly, the RVFV-like termination signal identified in the BUNV S segment is located potentially five nucleotides downstream of the mRNA 3′ end (if mRNA terminates at position 861) as determined in this work (Fig. 5). The RVFV-like termination signal overlaps the critical six residues in T1 (Fig. 6), but only shares two residues with the T1/T2 conserved pentanucleotide (Barr *et al.*, 2006), and is the region lacking in the S segment of virus rBUNVSΔT1. This suggests that transcription termination of S segment mRNAs from BUNV and RVFV, from two distinct genera in the family *Bunyaviridae*, may actually be signalled by a similar sequence. The observed redundancy of transcription termination for viral fitness in cell culture was surprising and further results from experiments in interferon-competent mammalian cells and in insect cells are required to explain the significance of mRNA transcription termination in the virus life cycle.

## Methods

### 

#### Cells and viruses.

BHK-21 cells were maintained in Glasgow modified Eagle’s medium supplemented with 10 % newborn calf serum and 5 % tryptose phosphate broth. Vero cells were grown in Dulbecco’s modified Eagle’s medium supplemented with 10 % FCS. Recombinant Bunyamwera viruses with nucleotide deletions in the 5′ UTR of the S genome segment were rescued as described previously (Lowen & Elliott, 2005; Lowen *et al.*, 2004).

#### Plaque assay.

Supernatants of virus-infected BHK-21 cells were diluted in PBS containing 2 % FCS and subsequently used to infect confluent Vero cells grown in six-well plates. After 1 h incubation at 37 °C, the cells were overlaid with MEM containing 2 % FCS and 1 % agarose, and incubated at 37 °C for 5 days. Cells were fixed with 4 % formaldehyde in PBS and plaques revealed with Giemsa stain.

#### Northern blotting.

Subconfluent BHK-21 cells were infected at an m.o.i. of 0.01 or left uninfected (mock) and total cell RNA was harvested 48 h later using the RNeasy RNA purification kit (Roche). Three micrograms of RNA was separated in a 1.8 % agarose gel and blotted onto a positively charged nylon membrane (Masek *et al.*, 2005). BUNV-specific RNAs were detected using digoxigenin-labelled probes complementary to either the genome (+ve sense probe) or the antigenome and mRNA (−ve sense probe) of the BUNV S segment. The probes covered the whole length of the BUNV S segment and were labelled by incorporation of UTP-digoxigenin (Roche) in a T7 polymerase-driven *in vitro* transcription reaction. Detection was carried out using an anti-digoxigenin antibody (Roche) according to the manufacturer’s instructions.

#### Western blotting.

Subconfluent BHK-21 cells were infected at an m.o.i. of 0.01 or left uninfected (mock). The cells were lysed 48 h post-infection in RIP buffer (50 mM Tris/HCl, 5 mM EDTA, 300 mM NaCl, 1 % Triton X-100) containing Complete protease inhibitor cocktail (Roche) and 25 U Benzonase (Merck) ml^−1^. Lysates were separated in 4–12 % gradient polyacrylamide gels (Invitrogen) and blotted onto nitrocellulose membrane (Amersham). Proteins were detected using antibodies directed against BUNV N (Leonard *et al.*, 2005) and tubulin (clone B512; Sigma).

#### 3′ RACE.

BHK-21 cells were infected at an m.o.i. of 5 or left uninfected (mock) and total cell RNA was isolated 18 h later using Trizol reagent (Invitrogen). Five micrograms of RNA was polyadenylated by 4 U poly(A) polymerase (Ambion) and then purified on RNeasy columns (Qiagen). First strand cDNA synthesis was carried out with one microgram polyadenylated RNA, primer RACE-OdT-AP (Roche) and 20 U Transcriptor reverse transcriptase (Roche). BUNV S segment-specific sequences were amplified by PCR including primers RACE-AP (Roche) and BUNS-707FW (5′-GTCTCTAGCTTAGGTTGG-3′), 1 µl cDNA and 5 U GoTaq polymerase (Promega). The PCR products were separated in a 2 % agarose gel and recovered with a commercial PCR product purification kit (Roche). In cases where the RT-PCR produced two distinct bands correlating to full-length RNA and mRNA (i.e. wtBUNV, rBUNVSΔCA and rBUNVSΔT2), only the shorter product was purified. Amplicons were TA-cloned into vector pGEM-T7 (Promega) and individual clones were selected for nucleotide sequencing.
